# Clinical utility of BOLD-MRI in accurate diagnosis and prognostic evaluation of diabetic nephropathy: a prospective renal biopsy-based cohort study

**DOI:** 10.1186/s13244-026-02274-9

**Published:** 2026-04-20

**Authors:** Qian Wang, Shaopeng Zhou, Yue Niu, Chaobo Li, Qiang Lyu, Pu Chen, Xiaojing Zhang, Lizhi Xie, Wanjun Shen, Yong Wang, Xueying Cao, Guangyan Cai, Xiangmei Chen, Haiyi Wang, Zheyi Dong

**Affiliations:** 1 Department of Nephrology, First Medical Center of Chinese PLA General Hospital, Nephrology Institute of the Chinese People’s Liberation Army, State Key Laboratory of Kidney Diseases, National Clinical Research Center for Kidney Diseases, Beijing Key Laboratory of Kidney Disease Research, Beijing, China; 2 Medical School of Chinese PLA, Beijing, China; 3 Department of Radiology, First Medical Center of Chinese PLA General Hospital, Beijing, China; 4 GE Healthcare, MR Research China, Beijing, China

**Keywords:** BOLD-MRI, Diabetic nephropathy, Differential diagnosis, Prognostic marker, R2*

## Abstract

**Objectives:**

To validate blood oxygen level-dependent MRI (BOLD-MRI) for non-invasive discrimination of diabetic nephropathy (DN) vs non-diabetic renal disease (NDRD) and prediction of end-stage renal disease (ESRD) in diabetic kidney disease (DKD).

**Materials and methods:**

A prospective cohort of 133 biopsy-proven DKD patients underwent BOLD-MRI. The semi-automated 12-layer concentric-objects method was used to analyze BOLD-MRI variables. Prognostic markers for ESRD were identified using univariate and multivariate Cox regression. Feature importance was used to select key diagnostic variables and establish logistic regression and machine-learning differential diagnosis models.

**Results:**

Among 133 patients (44 DN, 55 NDRD, 34 combined), 20 (15.5%) progressed to ESRD over a mean of 21.8 months. Higher renal medullary R2* (MR2*) (> 24 1/s) reduced ESRD risk by 52% (HR, 0.48) in DKD. Prognostic models integrating pathological grouping, hemoglobin levels, and cysC levels achieved a *c*-index of 0.90. For the DN and combined groups, MR2*, glomerular grading, interstitial lesions, interstitial fibrosis, and tubular atrophy were predictive of ESRD, with a *c*-index of 0.91. For differential diagnosis, the random forest (RF) model achieved an AUC of 0.901, with diabetic retinopathy, diabetes duration, albumin, blood urea nitrogen, MR2*, hypertension, and glycosylated hemoglobin as the most contributing factors. For the combined group classified as DN, the AUC of the RF model was 0.791; when classified as NDRD, the AUC was 0.856.

**Conclusion:**

MR2* shows potential value as a non-invasive diagnostic and prognostic tool in the assessment of DKD. However, BOLD-MRI remains a promising yet exploratory technique that requires external validation and interventional studies before clinical implementation.

**Critical relevance statement:**

Blood oxygen level-dependent-MRI-derived renal medullary R2* robustly predicts ESRD risk and distinguishes DN without biopsy, offering an immediately translatable, non-invasive biomarker for the precision management of DKD in routine nephrology practice.

**Trial registration:**

ClinicalTrials.gov, NCT03865914.

**Key Points:**

- Blood oxygen level-dependent-MRI medullary R2*(MR2*) > 24 s^−^^1^ halves DKD ESRD risk (HR 0.48).
- MR2* integrated with clinical variables drives *c*-index to 0.90 for ESRD prognosis.
- RF leveraging MR2* and clinical traits attains an AUC of 0.901 for diagnosing DN.

**Graphical Abstract:**

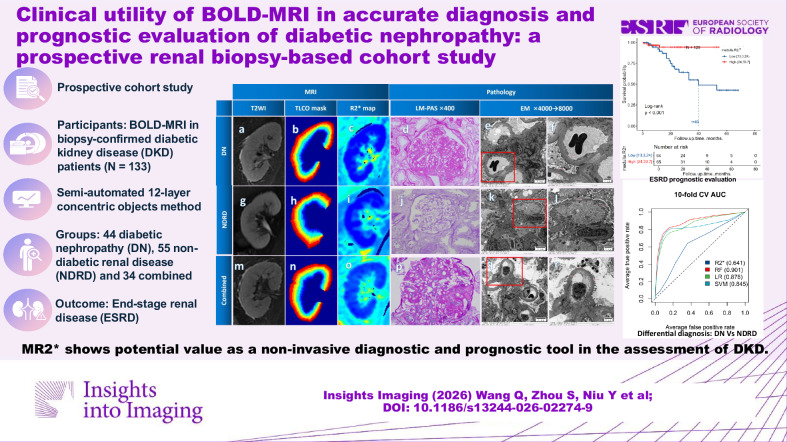

## Introduction

Diabetic nephropathy (DN), one of the most severe microvascular complications of diabetes mellitus [1], is characterized by a rising global prevalence due to the lack of early diagnostic tools and effective interventions [2]. As a leading cause of chronic and end-stage renal disease (ESRD) [3, 4], DN significantly contributes to mortality and disability in patients with diabetes [5–7]. Early non-invasive diagnosis and precise prognostic evaluation are critical for reducing the disease burden and guiding clinical decision-making. Early risk stratification in DN identifies rapid progressors sooner, enabling earlier intensive therapy, specialist referral, tailored follow-up, and trial enrollment. It reduces ESRD, helps patients understand their risk, and drives lifestyle changes and better adherence to RAS antagonists, finerenone, and sodium–glucose cotransporter-2 inhibitors (SGLT2i), while reducing overtreatment and optimizing resource use. However, current diagnostic strategies and prognostic tools for DN are inadequate, as they primarily rely on traditional biomarkers such as urinary protein and estimated glomerular filtration rate (eGFR), which fail to accurately assess early renal injury, disease progression, or long-term outcomes [8, 9]. Although renal biopsy remains the gold standard, its invasive nature limits its widespread or repeated application in DN management [10], underscoring the urgent need for non-invasive biomarkers to improve diagnostic and prognostic accuracy.

Advances in functional magnetic resonance imaging, particularly blood oxygen level-dependent MRI (BOLD-MRI), offer promising potential for the non-invasive evaluation of renal pathophysiology [11, 12]. BOLD-MRI can indirectly assess tissue oxygenation by detecting magnetic field disturbances caused by paramagnetic deoxyhemoglobin [13], providing insights into renal hypoxia, a key driver of diabetic kidney disease (DKD) progression under the “chronic hypoxia hypothesis” [14]. The relaxation rate (R2*), which is inversely correlated with tissue oxyhaemoglobin levels [15], serves as a biomarker for renal oxygenation. Elevated cortical R2* (CR2*) reflects reduced oxygenation, a hallmark of chronic kidney disease (CKD) progression [16]. Notably, renal R2* negatively correlates with eGFR, whereas reduced medullary R2* (MR2*) is associated with accelerated annual eGFR decline in moderate CKD [17]. Importantly, MR2* demonstrates sensitivity in detecting medullary hypoxia even in early-stage DN [18] and correlates with histopathological features such as interstitial fibrosis and tubular atrophy (IFTA) [19, 20].

Despite these advances, evidence supporting the role of BOLD-MRI in longitudinal prognosis and histopathological correlation in biopsy-confirmed DN cohorts remains limited. This study aimed to validate BOLD-MRI for non-invasive discrimination of DN vs non-diabetic renal disease (NDRD) and prediction of ESRD in DKD.

## Materials and methods

### Study participants

This single-center prospective cohort study was conducted from April 2018 to September 2023 at the Department of Nephrology, First Medical Center of the Chinese PLA General Hospital. A total of 140 patients with type 2 diabetes mellitus (T2DM) and kidney disease who underwent renal biopsy and BOLD-MRI examination were enrolled. All adult patients with biopsy results were eligible for BOLD-MRI examination, which was performed within 1 week after biopsy.

**Fig. 1:**
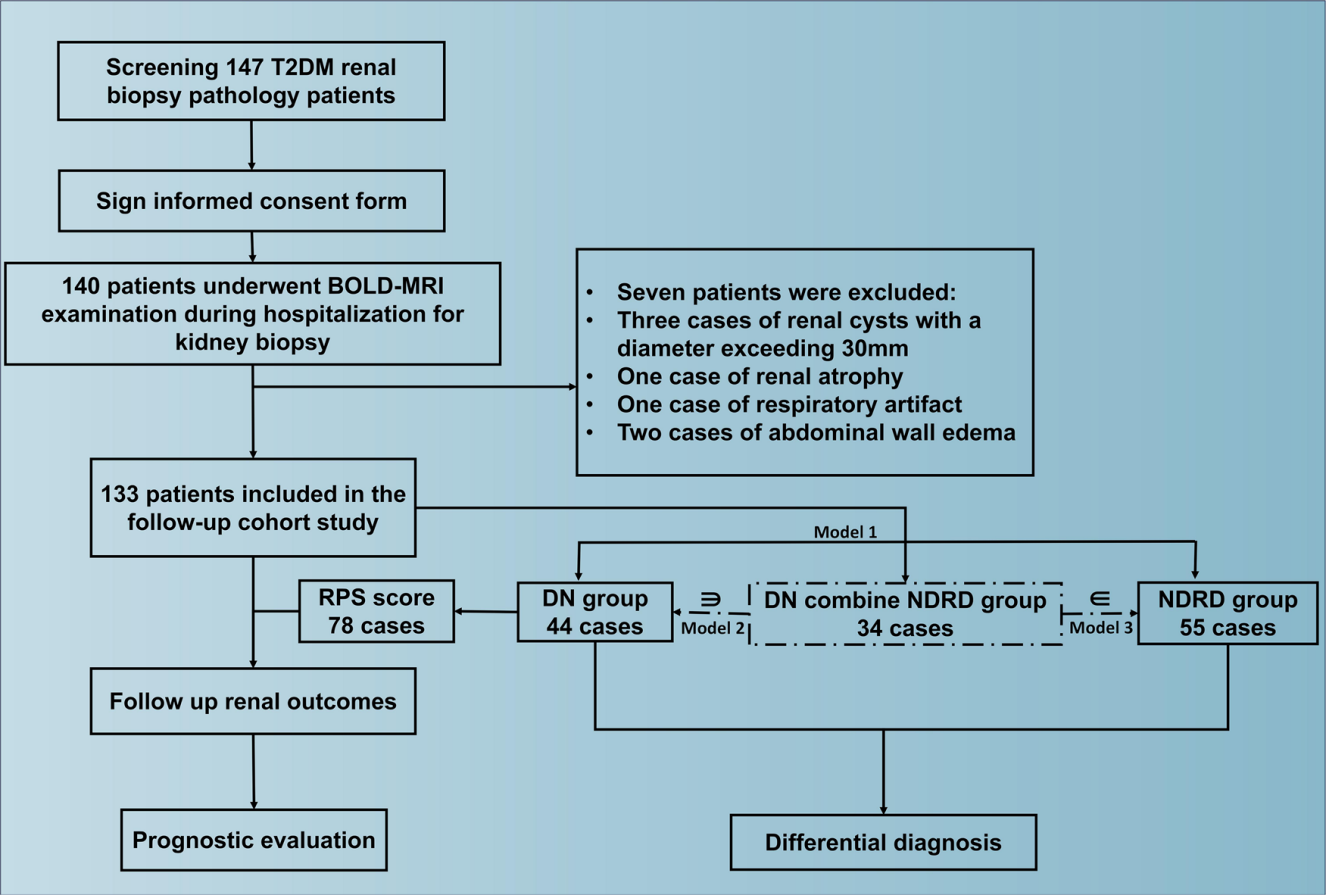
Flowchart showing the procedure for the selection of study participants. DN, diabetic nephropathy; NDRD, non-diabetic renal disease

Inclusion criteria were: (1) age 18–70 years; (2) diagnosis of T2DM; (3) renal impairment, albumin (Alb) excretion > 30 mg/day or GFR < 60 mL/min/1.73 m^2^, or both, of > 3 months’ duration; (4) renal biopsy and BOLD-MRI performed during the same hospitalization; and (5) provision of informed consent. The exclusion criteria were as follows: (1) renal tumors, large renal cysts (maximum diameter ≥ 30 mm), abnormal renal location, congenital renal dysplasia, or renal atrophy; (2) inadequate image quality for analysis due to severe respiratory motion artifacts; (3) significant abdominal edema or exudation; and (4) conditions affecting renal perfusion (e.g., renal artery stenosis or heart failure). This study was approved by the Research Ethics Committee of the Chinese People’s Liberation Army General Hospital (No.S2017-133-01), and informed consent was obtained from all participants. Follow-up was conducted in July 2024 to confirm renal outcomes; the renal endpoints were ESRD or all-cause death. A flowchart of the study is shown in Fig. 1.

### Renal biopsy and pathological evaluation scoring

All patients provided written informed consent before undergoing renal biopsy. Biopsies were performed by an experienced physician, and all specimens were independently reviewed by two pathologists. Any discrepancies were resolved through discussion until a consensus was reached. Tissue was processed and allocated for immunofluorescence, light microscopy, and electron microscopy. DN was diagnosed based on characteristic histological features, including glomerular hypertrophy, thickening of glomerular capillary basement membranes, diffuse mesangial expansion, nodular mesangial sclerosis (Kimmelstiel–Wilson nodules), exudative lesions such as capsular drop or fibrin cap, mesangiolysis, capillary microaneurysms, and hyalinosis of afferent and efferent arterioles [17]. NDRD was diagnosed based on classical criteria [18].

**Fig. 2:**
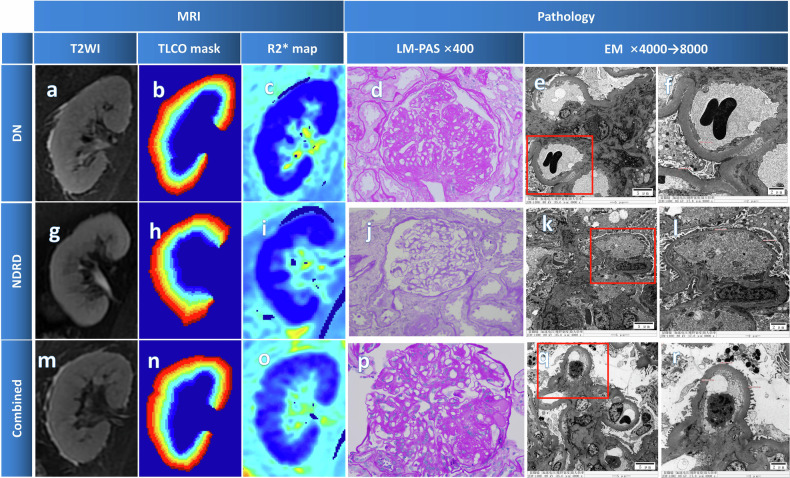
Comparison of MRI and pathological feature images. **a**–**f** Are from a 41-year old man with DN; **g**–**l** are from a 59-year-old woman with focal segmental glomerulosclerosis; **m**–**r** are from a 42-year-old man with DN combined with IgAN.The outer border (red) and inner border (blue) of renal tissue were defined by the operator on the R2* map for TLCO analysis. The color map of the 12 layers computed by the algorithm. DN:LM: the glomeruli exhibit focal, segmental, and mild mesangial cell proliferation, along with diffuse, global, and moderate mesangial matrix expansion. Focal, segmental, and mild endothelial cell proliferation is observed. K-W nodular lesions and exudative changes are present. Some capillary loops appear shrunken and poorly patent. The Bowman’s capsule wall shows diffuse thickening. EM: Significant thickening of glomerular basement membrane (GBM) (900–1097 nm). NDRD: LM: The glomeruli show no significant mesangial cell proliferation or mesangial matrix expansion. Endothelial cell proliferation is not evident, and capillary loops are patent. Parietal epithelial cells show no proliferation, and the Bowman’s capsule wall exhibits segmental thickening. EM: GBM shows no significant thickening (302–307 nm), and podocyte foot processes exhibit segmental effacement. Combined: LM: the glomeruli display focal, segmental, and mild mesangial cell proliferation, along with diffuse, segmental, and moderate mesangial matrix widening. Focal, segmental, and mild endothelial cell proliferation is observed. Mesangiolysis, capillary loop microaneurysms, and exudative changes are present. Some capillary loops are poorly patent, and the Bowman’s capsule wall shows segmental thickening and layering. EM: mesangial cell proliferation and mesangial matrix expansion are evident, with electron-dense deposits in the mesangial region. Capillary loops are poorly patent. Endothelial cell proliferation and segmental widening of the subendothelial space are observed. GBM is significantly thickened (672–804 nm), and podocytes exhibit extensive foot process effacement. LM, light microscopy; EM, electron microscopy

Pathological findings were evaluated according to the Pathologic Classification of DN [21]. Patients with DN were scored using the Renal Pathology Society (RPS) classification to assess the severity of glomerular classification (GC), interstitial lesions (IL; including IFTA and interstitial inflammation [II]), and vascular lesions (VL; including arteriolar hyalinosis [AH] and arteriosclerosis [AS; scored on the worst artery]) [21] (Fig. 2).

### Clinical and laboratory indexes

Demographic and clinical characteristics of the patients were recorded, including sex, age, diabetes duration, presence of diabetic retinopathy (DR), body mass index (BMI), presence of hypertension, systolic blood pressure (SBP), and diastolic blood pressure. Blood pressure was calculated as the mean of measurements obtained in the fasting and resting states on the day of the BOLD-MRI examination. Laboratory parameters, including fasting blood glucose, glycated hemoglobin (HbA1c), serum creatinine (Scr), blood urea nitrogen (BUN), serum uric acid, cystatin C (Cys-C), hemoglobin (Hb), hematocrit, Alb, triglycerides (TG), total cholesterol (TC), low-density- lipoprotein, proteinuria, 24-h urinary protein (24hUPro), and the presence or absence of glomerular hematuria, were measured within one week prior to MRI scanning. The eGFR was calculated using the 2009 CKD Epidemiology Collaboration (CKD-EPI) creatinine equation, which was also used to stage CKD [19]. The latest Scr levels were recorded during follow-up to assess renal outcomes.

### MRI image acquisition and analysis

All participants were required to fast for at least 4 h and undergo rigorous breathing training before MRI scanning. Imaging was performed with an eight-channel body array coil (3.0-T MRI Discovery 750; GE Medical Systems). A multiple echo FSPGR sequence (8 echoes ranging from 2.5 to 30 ms, field of view 300 × 300 mm, repetition time 100 ms, bandwidth 31.25 Hz/pixel, flip angle 35°, matrix 192 × 160, number of slices 9, slice thickness 7 mm) was used to acquire transverse relaxation (T2*)-weighted images in the oblique-coronal plane during breath-hold at end-expiration. T2* maps with the corresponding R2* (= 1/T2*) were calculated by fitting the pixel intensity at different echo times to an exponentially decaying function.

An internally modified version of the public program (https://github.com/bmilani/conObj) was used to generate the R2* images. The “12-layer concentric objects” technique was applied for BOLD-MRI analysis, segmenting the renal parenchyma into 12 equally thick layers extending from the cortex to the medulla and calculating the average R2* value for each layer within the kidney [22]. The outer and inner boundaries of the renal parenchyma were manually drawn (by a radiologist with approximately 3 years of experience) on the slice, offering the best visualization of the entire renal parenchyma from the R2* map using custom Matlab R2023a (Mathworks) (Fig. 2). To assess inter-observer reliability, 50% of the cases were randomly selected and independently delineated by a second radiologist with approximately 5 years of experience.

### Statistical analysis

All statistical analyses were performed using R software (version 4.4.0; R Foundation for Statistical Computing). Categorical variables were expressed as frequencies and proportions, while continuous variables were described as means and standard deviations for normally distributed data and medians with interquartile ranges for non-normally distributed data. Differences between groups were assessed using the chi-square, *t*-test, or Mann–Whitney *U*-test, as appropriate. Spearman’s rank correlation coefficient was used to evaluate correlations between variables. Missing data were handled with multiple imputations using the *mice* package (version 3.16.0). For survival analysis, two datasets were used: one with complete outcome data and another excluding patients with NDRD to include additional pathological scores. Prognostic Cox models were constructed using stepwise regression based on the Akaike Information Criterion and validated through ten-fold cross-validation, with the *c*-index reported for predictive performance. Cox regression was conducted using the *survival* package (version 3.7-0). Three models were developed for DN diagnosis: logistic regression, support vector machine (SVM), and random forest (RF), with variable selection based on the importance of the RF algorithm. Model performance was evaluated using the receiver operating characteristic (ROC) curve and the area under the curve (AUC), with a higher AUC indicating better diagnostic efficacy. Diagnostic modeling was performed using R packages *e1071* (version 1.7-14), *RF* (version 4.7-1.1), and *cvAUC* (version 1.1.4).

## Results

### Clinical characteristics

#### Patient demographics, clinical features, and pathology findings

According to the research flow chart (Fig. 1), 133 patients with T2DM who underwent renal biopsy were included in this study for clinicopathological characterization and BOLD-MRI parameter analysis. In the study cohort, 74.4% (*n* = 99) were men, with a mean age of 51.99 ± 10.0 years. The median duration of diabetes was 96.97 months. Regarding complications, 32.3% (*n* = 43) had DR, 70.5% (*n* = 93) had hypertension, and 43.6% (*n* = 58) had massive proteinuria (urinary protein > 3.5 g/24 h). Renal function assessment showed that 79.7% (*n* = 106) of patients were in stages 1–3 of CKD, with a baseline eGFR of 62.68 ± 18.35 mL/min/1.73 m^2^. For the BOLD-MRI parameters, the median CR2* was 19.56/s (IQR, 18.08–21.77), and the mean medullary R2* was 23.88 ± 3.88/s. For inter-observer reproducibility, excellent agreement was achieved for CR2* (ICC, 0.965; 95% CI: 0.943–0.978) and medullary R2* (ICC, 0.978; 95% CI: 0.964–0.986) (Supplement Table 1).

**Fig. 3:**
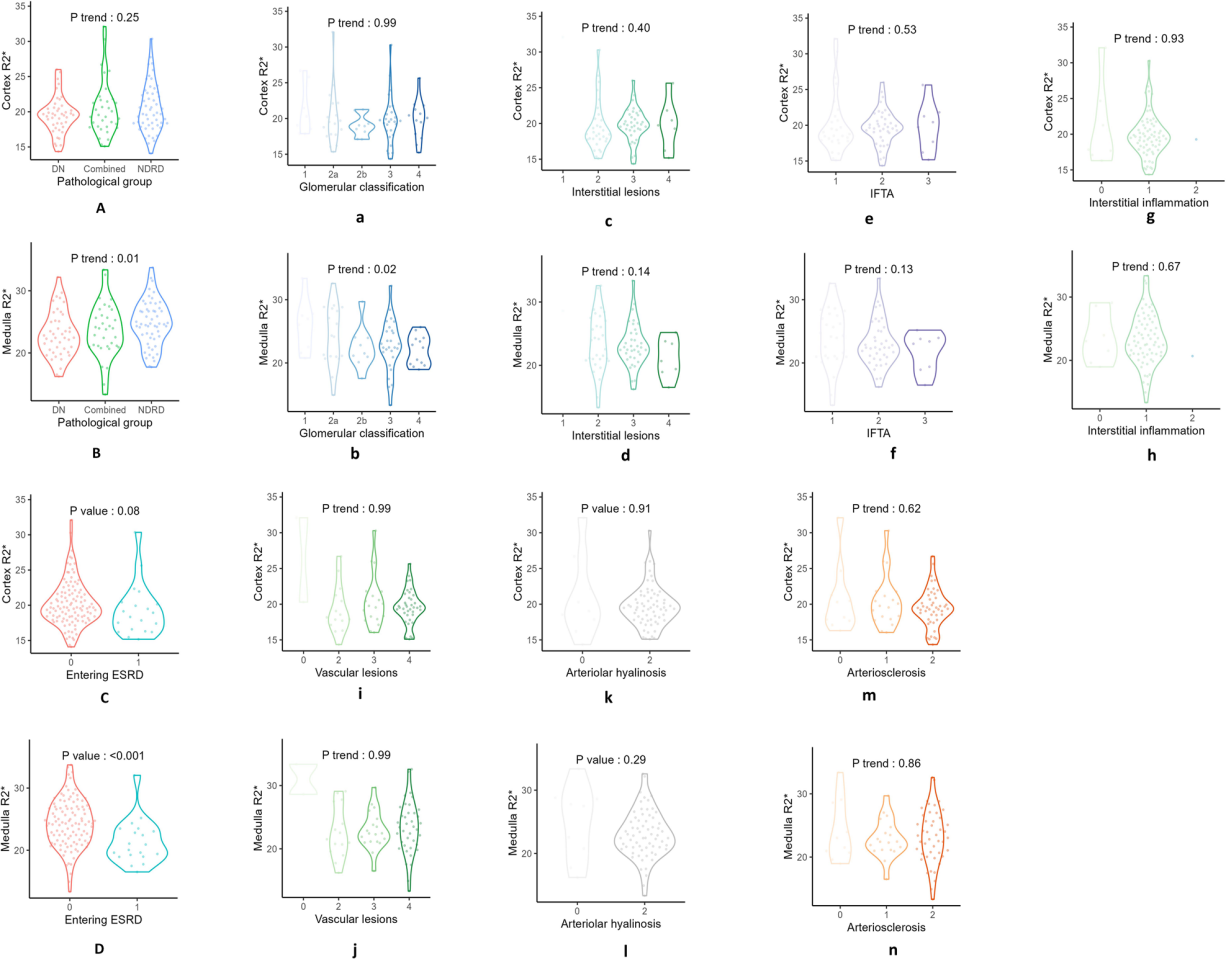
Violin plot of BOLD parameters with different pathological and renal outcomes. **A**, **B** Pathological group. **C**, **D** Renal outcome: entering ESRD or not. **a**–**n** Renal Pathological Score (RPS)–based stratification: Glomerular classification, Interstitial lesions, Interstitial fibrosis and atrophy (IFTA), Interstitial inflammation, Vascular lesions, Arteriolar hyalinosis, and Arteriosclerosis

The pathological classification showed that there were 44 cases (33.1%) in the DN group, 55 cases (41.4%) in the NDRD group, and 34 cases (25.6%) in the combined group. There were significant differences among the three groups in terms of parameters such as the course of diabetes, incidence of retinopathy, HbA1c, degree of hematuria, renal function indicators (blood creatinine, urea nitrogen, eGFR), Cys-C, TC, Hb, electrolyte (phosphorus, sodium, potassium), and renal MR2* value (*p* < 0.05) (Table 1 and Fig. 3).

**Table 1:** Clinical characteristics and laboratory data of patients

Parameter	Overall	DN	NDRD	DN combine NDRD	*p* value
*N*	133	44	55	34	
Sex (male), *N* (%)	99 (74.4)	33 (75.0)	38 (69.1)	28 (82.4)	0.377
Age, year, mean ± SD	51.99 ± 10.00	51.11 ± 9.76	52.62 ± 10.39	52.12 ± 9.89	0.758
BMI, kg/m^2^, mean ± SD	26.43 ± 3.39	26.32 ± 3.37	26.61 ± 3.83	26.27 ± 2.69	0.878
SBP, mmHg, mean ± SD	136.60 ± 21.65	140.11 ± 24.55	136.60 ± 20.33	132.06 ± 19.37	0.27
DBP, mmHg, median [IQR]	82.00 [70.00, 91.00]	83.00 [71.50, 93.00]	81.00 [71.50, 91.50]	82.00 [68.50, 91.00]	0.89
Diabetes duration, months, median [IQR]	96.97 [25.17, 158.60]	123.32 [75.67, 240.18]	25.23 [4.00, 98.50]	111.40 [63.00, 144.43]	< 0.001
DR, *N* (%)	43 (32.3)	29 (65.9)	3 (5.5)	11 (32.4)	< 0.001
hypertension, *N* (%)	93 (70.5)	36 (81.8)	33 (61.1)	24 (70.6)	0.082
Drinking, *N* (%)	52 (40.0)	17 (40.5)	18 (33.3)	17 (50.0)	0.298
Smoking, *N* (%)	59 (45.4)	22 (52.4)	24 (44.4)	13 (38.2)	0.461
HbA1c, %, median [IQR]	6.80 [6.20, 7.95]	7.85 [6.58, 8.88]	6.55 [6.20, 7.20]	6.50 [6.00, 7.60]	0.001
HbA1c ≥ 7, %, *N* (%)	55 (44.7)	30 (71.4)	14 (29.2)	11 (33.3)	< 0.001
FBG, mmol/L, median [IQR]	6.15 [5.18, 7.43]	6.58 [5.20, 8.79]	6.15 [5.34, 7.19]	5.78 [4.61, 6.86]	0.072
Scr, umol/L, median [IQR]	103.80 [80.50, 163.80]	130.45 [100.50, 180.38]	93.30 [71.25, 152.30]	94.25 [76.82, 129.38]	0.005
eGFR, mL/min/1.73 m^2^, median [IQR]	62.68 [33.81, 90.60]	53.26 [30.52, 68.88]	68.96 [46.24, 97.89]	74.25 [48.91, 93.46]	0.01
BUN, mmol/L, median [IQR]	7.48 [5.55, 10.45]	8.80 [7.40, 12.39]	6.14 [4.48, 10.23]	6.92 [5.56, 9.35]	0.001
Ua, mol/L, mean ± SD	376.91 ± 97.40	375.62 ± 82.91	379.41 ± 120.54	374.55 ± 72.04	0.971
Cys-C, mg/L, median [IQR]	1.23 [1.00, 1.73]	1.46 [1.21, 2.28]	1.13 [0.85, 1.41]	1.18 [1.02, 1.58]	0.003
CKD stage, *N* (%)					0.037
1	34 (25.6)	6 (13.6)	19 (34.5)	9 (26.5)	
2	37 (27.8)	9 (20.5)	16 (29.1)	12 (35.3)	
3	35 (26.3)	18 (40.9)	7 (12.7)	10 (29.4)	
4	18 (13.5)	7 (15.9)	9 (16.4)	2 (5.9)	
5	9 (6.8)	4 (9.1)	4 (7.3)	1 (2.9)	
Gh, N (%)	56 (42.1)	21 (47.7)	28 (50.9)	7 (20.6)	0.012
24hUPro, g/24 h, median [IQR]	2.93 [1.24, 5.37]	3.33 [1.58, 4.94]	2.38 [1.23, 5.78]	2.93 [1.15, 5.94]	0.902
TC, mmol/L, median [IQR]	4.77 [3.88, 6.00]	4.32 [3.71, 5.19]	5.05 [4.19, 6.31]	5.07 [3.67, 6.22]	0.046
TG, mmol/L, median [IQR]	2.08 [1.61, 2.84]	1.94 [1.61, 2.58]	2.35 [1.68, 3.11]	1.98 [1.42, 2.82]	0.265
LDL, mmol/L, median [IQR]	2.95 [2.18, 4.01]	2.68 [2.02, 3.62]	3.09 [2.44, 4.11]	3.28 [2.14, 4.36]	0.079
HDL, mmol/L, median [IQR]	1.00 [0.87, 1.14]	0.97 [0.90, 1.10]	1.03 [0.85, 1.16]	1.00 [0.87, 1.15]	0.957
TP, g/L, mean ± SD	57.69 ± 11.29	59.74 ± 8.42	56.68 ± 12.59	56.66 ± 12.22	0.255
ALB, g/L, median [IQR]	34.30 [25.80, 40.40]	35.90 [31.60, 38.73]	33.60 [23.95, 41.15]	34.55 [23.13, 41.20]	0.583
TIBC, mol/L, mean ± SD	41.38 ± 9.83	41.96 ± 8.73	41.72 ± 11.50	40.14 ± 8.55	0.722
SI, µmol/L, median [IQR]	13.20 [9.25, 16.75]	9.90 [8.50, 14.10]	15.20 [10.60, 18.00]	14.50 [12.00, 17.00]	0.019
SF, g/L, median [IQR]	273.25 [114.15, 440.20]	328.15 [236.20, 481.12]	232.80 [116.10, 427.80]	119.50 [77.15, 418.00]	0.06
RBC, g/L, mean ± SD	4.22 ± 0.71	3.94 ± 0.70	4.37 ± 0.68	4.31 ± 0.71	0.008
Hb, g/L, mean ± SD	126.23 ± 22.18	116.45 ± 21.44	130.78 ± 21.13	131.53 ± 21.16	0.002
MCH, pg, mean ± SD	30.05 ± 1.60	29.63 ± 1.36	30.05 ± 1.82	30.60 ± 1.39	0.012
MCHC, g/L, median [IQR]	344.50 [336.00, 351.25]	344.50 [336.00, 351.00]	343.00 [334.00, 352.50]	346.00 [338.00, 352.00]	0.665
HCT, %, median [IQR]	0.35 [0.23, 0.40]	0.34 [0.29, 0.37]	0.35 [0.13, 0.39]	0.38 [0.26, 0.42]	0.302
MCV, fL, median [IQR]	86.35 [84.38, 89.53]	85.80 [84.00, 89.00]	86.20 [84.35, 89.65]	87.90 [85.20, 89.80]	0.327
Hp, mg/dL, (median [IQR])	130.00 [71.80, 178.50]	149.00 [116.00, 193.00]	131.50 [89.65, 172.75]	70.10 [29.02, 128.50]	< 0.001
Na, mmol/L median [IQR]	141.10 [139.50, 142.60]	141.30 [140.07, 142.72]	140.10 [138.38, 141.62]	141.40 [139.90, 142.80]	0.022
K, umol/L, median [IQR]	3.98 [3.68, 4.32]	4.14 [3.84, 4.47]	3.82 [3.66, 4.17]	3.87 [3.67, 4.22]	0.035
P, mmol/L, median [IQR]	1.28 [1.12, 1.38]	1.32 [1.23, 1.52]	1.23 [1.08, 1.34]	1.28 [1.07, 1.38]	0.021
cortex R2*, 1/s, median [IQR]	19.56 [18.08, 21.77]	19.53 [18.08, 20.48]	19.68 [18.23, 22.62]	19.41 [17.88, 21.61]	0.25
medulla R2*, 1/s, mean ± SD	23.88 ± 3.88	23.00 ± 3.63	24.77 ± 3.65	23.56 ± 4.32	0.01
Follow-up time, months, median [IQR]	15.00 [8.00, 30.00]	15.00 [8.00, 25.50]	14.00 [7.50, 29.50]	24.00 [8.25, 53.75]	0.318
Latest Scr, umol/L, median [IQR]	113.20 [82.10, 170.00]	139.30 [99.60, 300.00]	91.50 [70.42, 135.45]	119.35 [90.75, 145.27]	0.002
Latest eGFR, mL/min/1.73 m^2^, median [IQR]	62.92 [35.00, 88.89]	44.54 [19.13, 73.84]	78.43 [47.72, 96.23]	59.17 [44.63, 74.90]	0.007
Latest.24hUPro, g/24 h, median [IQR]	1.44 [0.65, 3.71]	2.95 [0.94, 3.71]	1.20 [0.85, 3.01]	1.23 [0.42, 4.50]	0.342
Endpoint, *N* (%)					
renal replacement therapy, *N* (%)	14 (10.5)	12 (27.3)	1 (1.8)	1 (2.9)	< 0.001
all cause mortality, *N* (%)	5 (3.8)	3 (6.8)	0 (0.0)	2 (5.9)	< 0.001
entering ESRD, *N* (%)	20 (15.5)	15 (34.9)	3 (5.6)	2 (6.2)	< 0.001

Data are presented as *n* (%), mean ± SD, or median (IQR)

*BMI* body mass index, *SBP* systolic blood pressure, *DBP* diastolic blood pressure, *DR* diabetic retinopathy, *HbA1c* glycated hemoglobin, *FBG* fasting blood glucose, *Scr* serum creatinine, *eGFR* estimated glomerular filtration rate, *BUN* blood urea nitrogen, *Ua* blood uric acid, *Cys-C* serum cystatin C, *CKD* chronic kidney disease, *Gh* glomerular hematuria, *24hUPro* 24-h urinary protein, *TC* total cholesterol, *TG* triglycerides, *LDL* low-density lipoprotein, *TP* total serum protein, *ALB* serum albumin, *TIBC* total iron binding capacity, *SI* serum iron, *SF* serum ferritin, *RBC* red blood cells, *Hb* hemoglobin, *MCH* mean hemoglobin content of red blood cell, *MCHC* mean corpuscular hemoglobin concentration, *HCT* hematocrit, *MCV* mean red blood cell volume, *Hp* haptoglobin, *Na* sodium, *K* potassium, *P* phosphorus

The median follow-up time was 15 months, with a mean follow-up of 21.8 months. At the last follow-up, the mean Scr level was 113.20 μmol/L, corresponding to a mean eGFR of 62.92 mL/min/1.73 m^2^. Endpoint analysis showed that 14 cases (10.5%) received renal replacement therapy, 5 cases (3.8%) experienced all-cause mortality, and 20 cases (15.5%) progressed to ESRD. There was a significant difference in the incidence of endpoint events among the three groups (DN group > combined group > NDRD group, *p* < 0.001) (Table 1). Further analysis of the clinicopathological characteristics of ESRD progressors showed that this subgroup had a longer duration of diabetes (*p* = 0.017), lower baseline eGFR (*p* < 0.001), more severe proteinuria (*p* = 0.01), more severe anemia (*p* < 0.001), and a lower renal MR2* value (20.48 [19.18–23.15] vs 24.31 [21.59–26.77], *p* < 0.001) (Table 2).

**Table 2:** Comparison of clinicopathological characteristics of patients entering ESRD or non-ESRD

Parameter	Overall	Non- ESRD	Entering ESRD	*p*
*N*	129	109	20	
Sex (male), *N* (%)	97 (75.2)	85 (78.0)	12 (60.0)	0.153
Age, year, mean ± SD	53.00 [45.00, 60.00]	53.00 [45.00, 60.00]	52.50 [44.75, 58.50]	0.81
BMI, kg/m^2^, mean ± SD	26.55 (3.36)	26.73 (3.40)	25.55 (2.97)	0.122
SBP, mmHg, mean ± SD	136.64 (21.92)	135.06 (21.46)	145.25 (22.93)	0.077
DBP, mmHg, median [IQR]	82.00 [70.00, 91.00]	81.00 [70.00, 91.00]	86.00 [76.75, 89.75]	0.283
Diabetes duration, months, median [IQR]	97.00 [24.97, 158.77]	88.43 [24.00, 151.77]	122.20 [91.40, 204.61]	0.017
DR, *N* (%)	42 (32.6)	31 (28.4)	11 (55.0)	0.038
hypertension, *N* (%)	90 (70.3)	74 (68.5)	16 (80.0)	0.444
Pathological group (%)				< 0.001
DN	43 (33.3)	28 (25.7)	15 (75.0)	
NDRD	54 (41.9)	51 (46.8)	3 (15.0)	
DN combine NDRD	32 (24.8)	30 (27.5)	2 (10.0)	
HbA1c, %, median [IQR]	6.80 [6.20, 7.90]	6.80 [6.20, 7.90]	6.75 [6.05, 8.07]	0.952
FBG, mmol/L, median [IQR]	6.13 [5.18, 7.43]	6.22 [5.27, 7.32]	5.42 [4.74, 7.71]	0.267
Scr, µmol/L, median [IQR]	103.80 [76.70, 168.10]	97.60 [75.40, 135.40]	183.30 [154.18, 304.03]	< 0.001
eGFR, mL/min/1.73 m^2^, median [IQR]	62.68 [33.81, 90.60]	68.47 [49.21, 95.86]	27.30 [18.92, 41.06]	< 0.001
BUN, mmol/L, median [IQR]	7.43 [5.52, 10.35]	6.99 [5.03, 9.47]	9.97 [8.82, 13.64]	< 0.001
Ua, mol/L, mean ± SD	375.99 (97.97)	381.26 (99.77)	347.29 (83.95)	0.117
Cys-C, mg/L, median [IQR]	1.23 [0.99, 1.68]	1.17 [0.94, 1.49]	2.27 [1.57, 2.83]	< 0.001
24 hUPro, g/24 h, median [IQR]	2.93 [1.24, 5.37]	2.38 [1.09, 5.10]	4.63 [3.21, 6.60]	0.01
TC, mmol/L, median [IQR]	4.76 [3.87, 6.00]	4.76 [3.87, 6.02]	4.90 [3.96, 5.90]	0.816
TG, mmol/L, median [IQR]	2.08 [1.58, 2.87]	2.10 [1.48, 2.90]	1.93 [1.68, 2.77]	0.961
ALB, g/L, median [IQR]	34.40 [26.60, 40.40]	35.80 [26.20, 41.20]	33.25 [27.52, 36.30]	0.149
SI, µmol/L, median [IQR]	13.20 [9.25, 16.75]	14.85 [11.12, 17.85]	8.60 [6.70, 10.70]	< 0.001
SF, g/L, median [IQR]	272.70 [114.80, 446.00]	242.90 [113.50, 427.80]	349.75 [152.20, 589.45]	0.137
Hb, g/L, mean ± SD	126.46 (22.28)	130.07 (20.88)	106.75 (19.54)	< 0.001
MCH, pg, mean ± SD	30.06 (1.61)	30.20 (1.66)	29.22 (0.96)	0.001
HCT, %, median [IQR]	0.44 [0.37, 13.00]	0.47 [0.38, 13.00]	0.34 [0.31, 10.19]	0.021
Hp, mg/dL, (median [IQR])	130.00 [72.40, 178.25]	129.00 [71.80, 166.50]	172.00 [96.90, 217.00]	0.139
Na, mmol/L, median [IQR]	141.05 [139.50, 142.60]	141.10 [139.15, 142.55]	141.00 [140.25, 143.30]	0.28
K, µmol/L, median [IQR]	4.01 [3.68, 4.33]	3.93 [3.68, 4.24]	4.36 [3.80, 4.62]	0.065
P, mmol/L, median [IQR]	1.27 [1.12, 1.38]	1.26 [1.09, 1.37]	1.31 [1.22, 1.44]	0.21
cortex R2*, 1/s, median [IQR]	19.55 [17.91, 21.67]	19.67 [18.27, 21.77]	18.64 [16.51, 20.21]	0.082
medulla R2*, 1/s, mean ± SD	23.96 [21.06, 26.67]	24.31 [21.59, 26.77]	20.48 [19.18, 23.15]	< 0.001

*24hUPro* 24-h urinary protein, *ALB* albumin, *BMI* body mass index, *BUN* blood urea nitrogen, *Cys-C* cystatin C, *DBP* diastolic blood pressure, *DN* diabetic nephropathy, *DR* diabetic retinopathy, *eGFR* estimated glomerular filtration rate, *FBG* fasting blood glucose, *Hb* hemoglobin, *HbA1c* glycated hemoglobin, *HCT* hematocrit, *Hp* haptoglobin, *K* potassium, *MCH* mean corpuscular hemoglobin, *Na* sodium, *NDRD* non-diabetic renal disease, *P* phosphorus, *SBP* systolic blood pressure, *Scr* serum creatinine, *SF* serum ferritin, *SI* serum iron, *TC* total cholesterol, *TG* triglycerides, *Ua* blood uric acid

#### Correlation analysis of clinicopathological features

Violin Plot Analysis (Fig. 3): analysis based on violin plots revealed that renal MR2* values revealed significant differences among ESRD progression status (*p* < 0.001) and pathological classification (DN group vs NDRD group vs combined group, *p* = 0.01). In contrast, the renal CR2* values did not show statistical significance among the groups (*p* > 0.05).

**Fig. 4:**
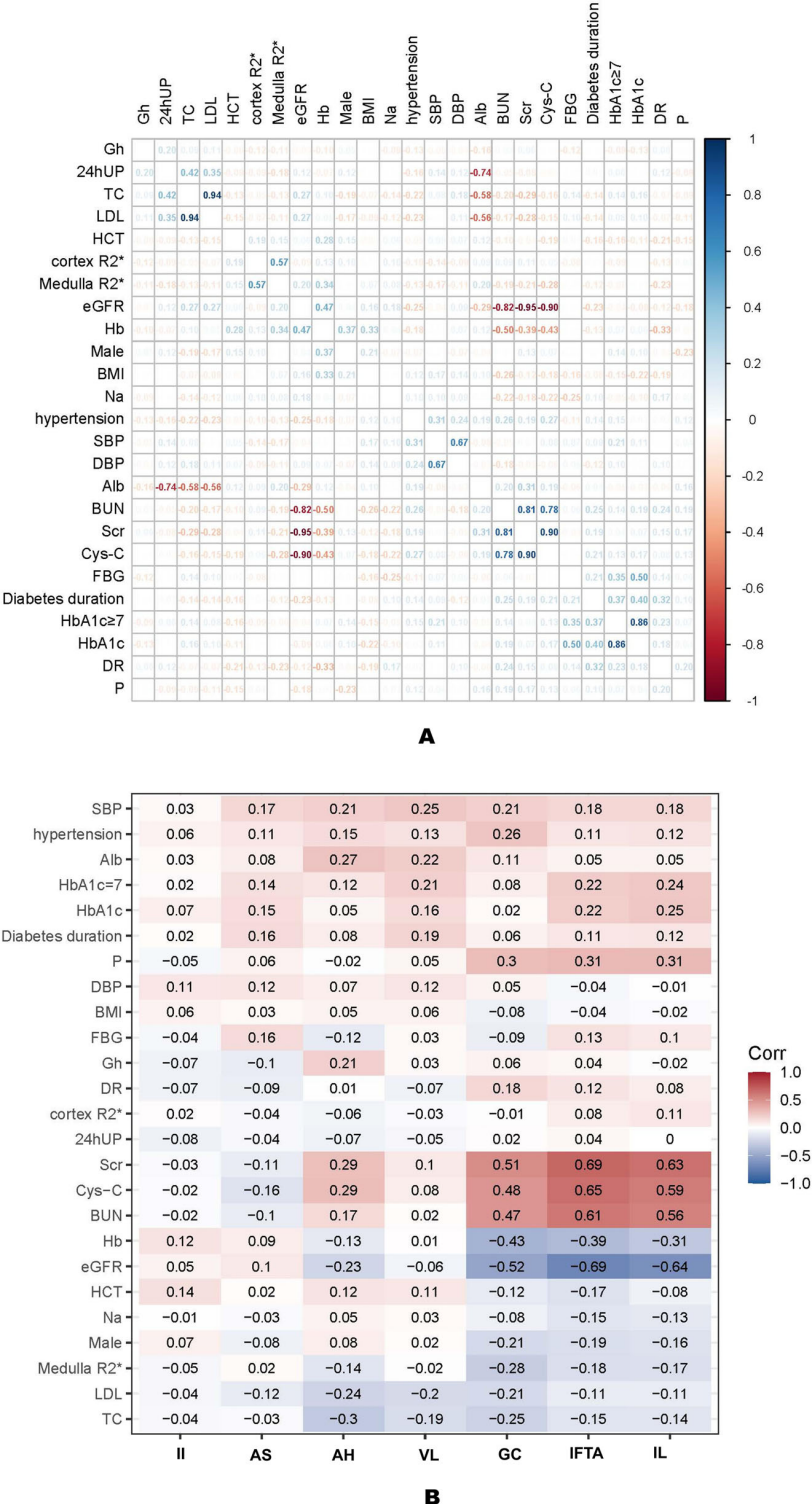
Correlation heatmap between BOLD parameters and Clinicopathological features. **A** Clinical parameter correlations in the DKD cohort (*n* = 133). **B** Clinicopathological analysis in non-Pure NDRD subcohort (*n* = 78). AH, arteriolar hyalinosis; AS, arteriosclerosis; GC, glomerular classification; IL, interstitial lesions; IFTA, interstitial fibrosis and tubular atrophy; II, interstitial inflammation; VL, vascular lesions

Spearman correlation analysis: to determine whether BOLD-MRI parameters independently predict renal prognosis from conventional clinical indicators, we conducted a Spearman correlation analysis on the clinical characteristics and R2* parameters of 133 patients. The results indicated that renal CR2* had no significant correlation with any clinical indicators (including eGFR, Scr, and proteinuria). Conversely, renal MR2* demonstrated a weak positive correlation with eGFR (*r* = 0.20, *p* = 0.02), a weak negative correlation with Cys-C (*r* = −0.28, *p* = 0.003), and a moderate positive correlation with Hb levels (*r* = 0.34, *p* < 0.001) (Table 3 and Fig. 4A).

**Table 3:** Correlation analysis between BOLD-R2* and clinicopathological parameters

Parameter	Cortex R2*		Medulla R2*	
	*r*	*p* value	*r*	*p* value
Male	0.1	0.28	0.01	0.93
Hypertension	−0.1	0.26	−0.13	0.93
BMI	0.01	0.88	0.07	0.13
Diabetes duration	0.02	0.86	−0.12	0.4
DR	−0.13	0.14	−0.23	0.18
SBP	−0.14	0.11	−0.17	0.009
24hUP	−0.09	0.33	−0.18	0.22
Gh	−0.12	0.18	−0.11	0.04
FBG	−0.08	0.37	−0.02	0.21
Alb	0.09	0.33	0.2	0.03
HbA1c	0.01	0.94	−0.01	0.93
Scr	0.11	0.21	−0.21	0.02
eGFR	−0.09	0.32	0.2	0.02
Cys-C	0.05	0.59	−0.28	0.003
TC	−0.05	0.53	−0.13	0.14
LDL	−0.07	0.44	−0.11	0.2
Hb	0.13	0.15	0.34	< 0.001
HCT	0.19	0.04	0.15	0.1
GC	−0.01	0.92	−0.28	0.01
IL	0.11	0.35	−0.17	0.13
IFTA	0.08	0.49	−0.18	0.12
II	0.02	0.89	−0.05	0.67
VL	−0.03	0.81	−0.02	0.87
AH	−0.06	0.58	−0.14	0.21
AS	−0.04	0.73	0.02	0.84

*24hUP* 24-h urinary protein, *AH* arteriolar hyalinosis, *Alb* albumin, *AS* arteriosclerosis, *BMI* body mass index, *Cys-C* cystatin C, *DR* diabetic retinopathy, *eGFR* estimated glomerular filtration rate, *FBG* fasting blood glucose, *GC* Glomerular classification, *Gh* glomerular hematuria, *Hb* hemoglobin, *HbA1c* glycated hemoglobin, *HCT* hematocrit, *IFTA* interstitial fibrosis and tubular atrophy, *IL* interstitial lesions, *II* interstitial inflammation, *LDL* low-density lipoprotein, *SBP* systolic blood pressure, *Scr* serum creatinine, *TC* total cholesterol, *VL* Vascular lesions

Further analysis in patients with non-pure NDRD: further analysis was conducted on the renal pathological RPS scores and imaging parameters (CR2* and MR2* values) of 78 patients with non-pure NDRD (DN and combined groups). The results showed weak correlations between renal CR2* and MR2* values and the seven pathological indicators. Specifically, MR2* values exhibited a negative correlation with glomerular grading (*r* = −0.28, *p* < 0.01). Notably, the degree of pathological tissue damage (including glomerular grading, IL, and IFTA) showed moderate correlations with clinical renal function indicators (eGFR and Cys-C) and the degree of anemia (Table 3 and Fig. 4B).

#### ESRD prognostic evaluation model

##### Prognostic model for the entire population cohort

**Fig. 5:**
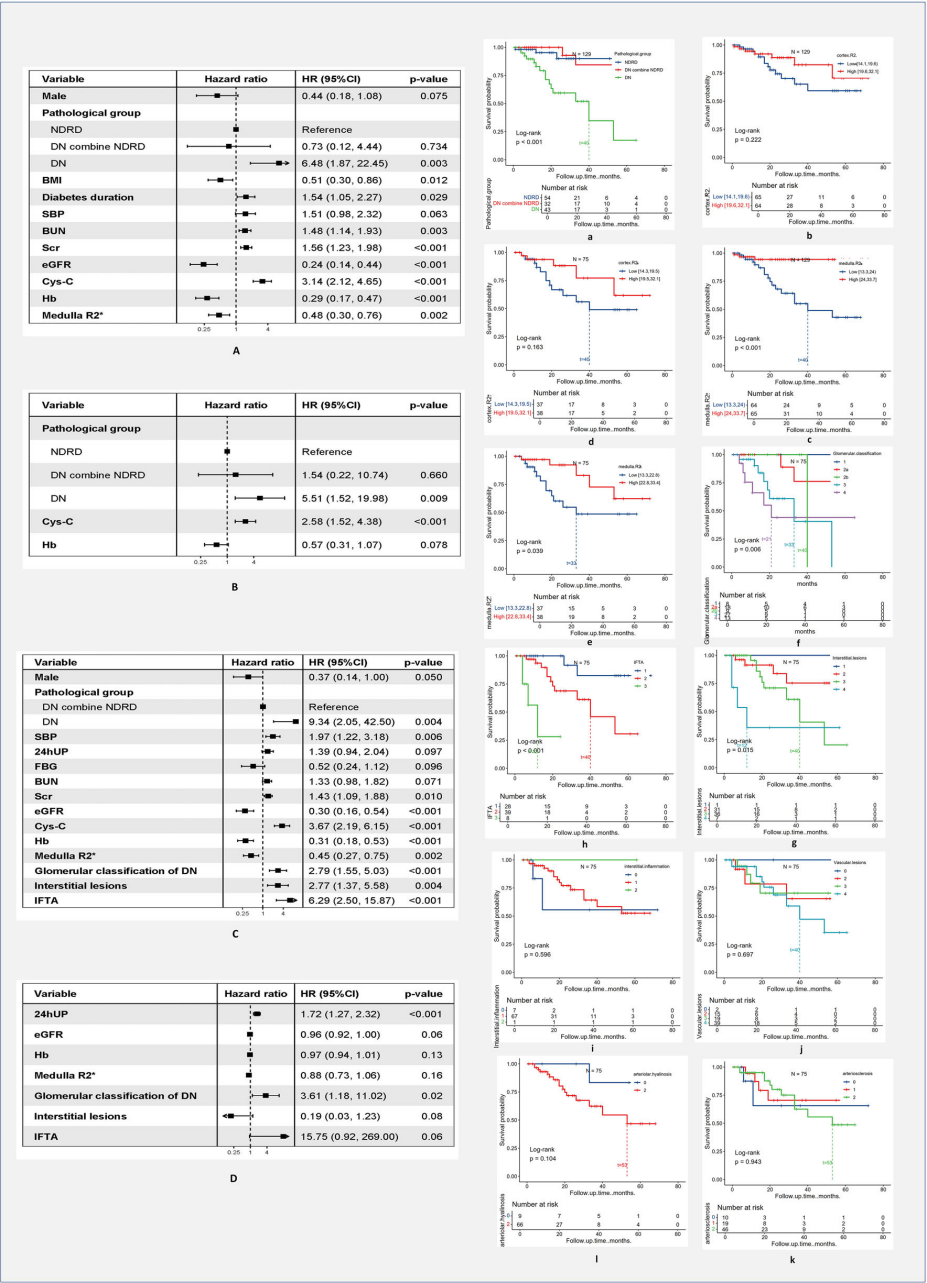
Univariate and multivariate Cox regression and KM Survival analysis of ESRD risk. **A**–**D** Univariate and multivariate Cox regression, **A** forest plot: univariate Cox regression analysis for the entire population, **B** forest plot: multivariate Cox regression analysis for the entire population, **C** Forest plot: univariate Cox regression analysis for the RPS assessment population, and **D** forest plot: multivariate Cox regression analysis for the RPS assessment population. **a**–**l** KM Survival analysis, **a**–**c** full data population, **d**–**I** RPS assessment population

Using Cox proportional hazards regression, we constructed an ESRD prognostic model for 129 patients with DKD (4 lost to follow-up). Kaplan–Meier univariate survival analysis showed that the survival curve of the DN group consistently lay below those of the NDRD and combined groups throughout the study period (Fig. 5a). The log-rank test indicated significant differences in survival times among the three groups (*p* < 0.001), with the DN group exhibiting the worst prognosis and a median survival of 40 months. Univariate Cox regression analysis revealed that patients with DN had a 6.48-fold higher risk of progressing to ESRD than those with NDRD (Fig. 5A). The survival curve of the higher MR2* group remained above that of the lower group during the study period, with significant differences in survival times (*p* < 0.001) (Fig. 5c). In contrast, CR2* did not provide prognostic information (Fig. 5b). Additionally, clinical parameters such as BMI, disease duration, SBP, renal function (eGFR, Scr, BUN, Cys-C), and Hb level were predictive of prognosis (*p* < 0.05) (Fig. 5A). Multivariate Cox proportional hazards regression analysis identified pathological grouping, Hb level, and Cys-C level as independent prognostic factors for renal survival in patients with DKD (Fig. 5B). Pathological grouping and Cys-C maintained statistical significance in the multivariate analysis, with a *c*-index of 0.90 from ten-fold cross-validation.

##### Prognostic model for DN and combined groups (RPS assessment population)

For the 75 patients in the DN and combined groups (with 3 lost to follow-up), we quantified pathological RPS scores and incorporated clinical and pathological parameters to construct a Cox proportional hazards regression model for ESRD progression in patients with non-pure NDRD. Univariate survival analysis demonstrated that MR2*, glomerular grading, IL, and IFTA were predictive of ESRD in pathologically diagnosed patients with DN, whereas other RPS scores lacked prognostic value (Fig. 5d–k). For every one-unit increase in glomerular grading, IL, and IFTA scores, the risk of ESRD increased by 2.79, 2.77, and 6.29 times, respectively (Fig. 5C). Multivariate Cox proportional hazards regression analysis indicated that 24hUPro, eGFR, Hb, MR2*, glomerular grading, IL, and IFTA were independent prognostic factors for renal survival in patients with DKD (Fig. 5D). The prognostic model had a *c*-index of 0.91 from ten-fold cross-validation.

### Differential diagnosis model

#### Comparison between DN and NDRD groups

Compared with patients in the NDRD group, those in the DN group had a longer duration of diabetes, a higher incidence of DR, higher HbA1c levels, better-controlled lipid profiles, lower estimated eGFR, and lower Hb levels. The renal MR2* was significantly lower in the DN group than in the NDRD group (23.00 ± 3.63 vs 24.77 ± 3.65, *p* = 0.018).

#### Univariate/multivariate analysis

To evaluate the independent contribution of Medulla R2* to differential diagnosis, we conducted univariate and multivariate logistic regression analyses (Supplementary Fig. 1). In univariate analysis (Panel A), Medulla R2* demonstrated a significant association with DKD status (OR 0.87, 95% CI: 0.77–0.98, *p* = 0.022), indicating its standalone discriminatory capability. However, in multivariate analysis adjusting for established clinical risk factors including DR, diabetes duration, HbA1c, hypertension, ALB, and BUN (Panel B), the effect estimate attenuated to non-significance (OR 0.94, 95% CI: 0.77–1.15, *p* = 0.571).

### Two-stage diagnostic performance of MR2* in differential diagnosis

**Fig. 6:**
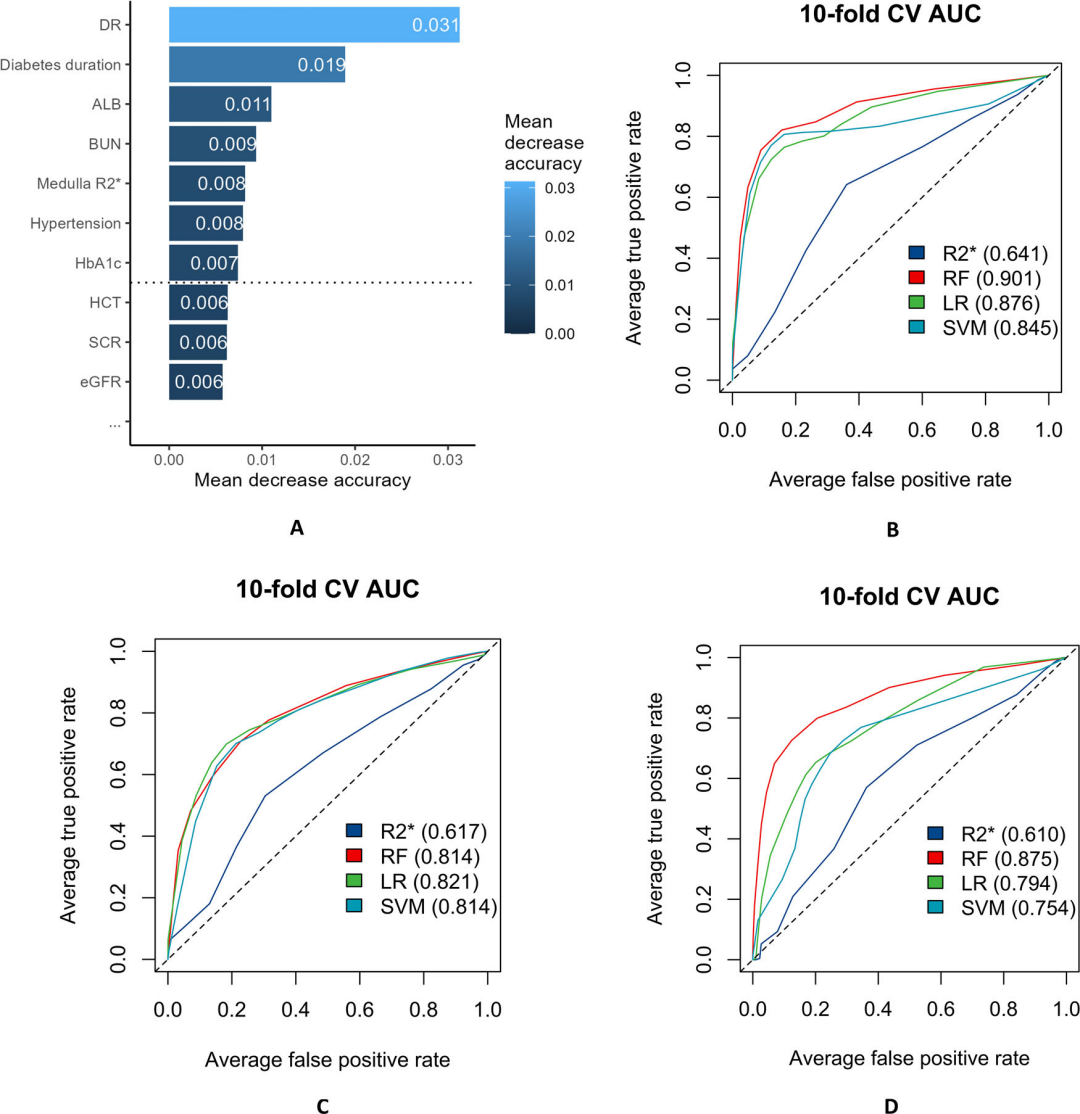
Differential diagnosis model and feature importance ranking based on BOLD-R2*. **A** Feature importance ranking. **B** Differential diagnosis cohort 1: DN Vs NDRD. **C** Differential diagnosis cohort 2: DN combine NDRD is classified into DN. **D** Differential diagnosis cohort 3: DN combine NDRD is classified into NDRD

To evaluate both the standalone and incremental diagnostic value of MR2*, we employed a two-stage modeling approach across three cohorts (Fig. 6). Feature importance of the RF model was used to identify parameters that could distinguish between DN and NDRD. Seven parameters were selected: DR, diabetes duration, Alb, BUN, renal MR2*, hypertension, and HbA1c (Fig. 6A).

Independent performance of MR2*: when used as the sole predictor, Medulla R2* demonstrated modest but consistent discriminatory capability across all three cohorts. The AUC values for MR2* alone were 0.64 in Cohort 1 (Fig. 6B), 0.617 in Cohort 2 (Fig. 6C), and 0.610 in Cohort 3 (Fig. 6D). These findings indicate that MR2* possesses intrinsic diagnostic information for distinguishing DKD, although its standalone performance is limited compared to comprehensive clinical assessment. The consistency of AUC values across diverse patient populations (range 0.610–0.641) supports the robustness and generalizability of this imaging biomarker.

Performance of integrated models: when MR2* was integrated with clinical variables using machine learning algorithms, diagnostic performance improved substantially across all cohorts. Diagnostic models were constructed using logistic regression (LR), SVM, and RF, and validated via ten-fold cross-validation. The AUC values for the models were RF (0.901), LR (0.876), and SVM (0.845). The RF model demonstrated the highest diagnostic efficacy, with an accuracy of 0.857, a sensitivity of 0.801, a specificity of 0.901, and an F1-score of 0.832 (Table 4 and Fig. 6B). When the combined group was classified as DN (Cohort 2), the AUC values were RF (0.791), LR (0.750), and SVM (0.764), with RF demonstrating the highest diagnostic efficacy. When the combined group was classified as NDRD (Cohort 3), the AUC values were RF (0.856), LR (0.786), and SVM (0.761) (Table 4 and Fig. 6C, D).

**Table 4:** Diagnostic efficacy of the differential diagnostic model between DN and NDRD

Model	Accuracy	Sensitivity	Specificity	Pos pred value	Neg pred value	F1	AUC
Model 1: DN Vs NDRD							
RF	0.857	0.801	0.901	0.866	0.850	0.832	0.901
LR	0.831	0.749	0.896	0.853	0.817	0.797	0.876
SVM	0.856	0.807	0.895	0.860	0.853	0.832	0.845
Model 2: DN combine NDRD is classified into DN							
RF	0.708	0.849	0.595	0.626	0.831	0.721	0.791
LR	0.718	0.780	0.669	0.653	0.792	0.711	0.750
SVM	0.710	0.726	0.698	0.658	0.761	0.690	0.764
Model 3: DN combine NDRD is classified into NDRD							
RF	0.743	0.889	0.695	0.485	0.951	0.628	0.856
LR	0.777	0.699	0.803	0.533	0.892	0.605	0.786
SVM	0.720	0.788	0.699	0.457	0.911	0.579	0.761

*DN* diabetic nephropathy, *LR* logistic regression, *NDRD* non-diabetic renal disease, *RF* random forest, *SVM* support vector machine

## Discussion

DKD is one of the leading causes of ESRD. Early diagnosis and accurate prognostic assessment are crucial for delaying disease progression. However, traditional clinical indicators, such as eGFR and proteinuria, have certain limitations for early diagnosis and prognostic evaluation [23]. In recent years, BOLD-MRI, as a non-invasive, contrast agent-free, and radiation-free imaging technique, has gradually demonstrated significant value in the early diagnosis and renal function assessment of CKD [24–26]. Through a prospective cohort design, this study systematically explored, for the first time, the clinicopathological correlations and prognostic diagnostic value of BOLD-MRI in DKD, filling a research gap in this field. Based on a cohort of 133 patients with T2DM confirmed by renal biopsy, this study found a significant association between MR2* and the progression of renal function, as well as pathological diagnosis. Furthermore, the study innovatively developed a multimodal prediction and diagnostic model integrating BOLD-MRI features, clinical indicators, and renal pathological parameters, overcoming the limitations of traditional single-biomarker assessments. This provides a clinically translatable new tool for non-invasive quantitative analysis of renal tissue hypoxia and individualized treatment decision-making.

BOLD-MRI exhibits excellent complementarity with traditional indicators, such as eGFR and proteinuria. While eGFR primarily reflects glomerular filtration function, and proteinuria is influenced by multiple factors, both have limited sensitivity in early DKD and fail to reflect renal oxygenation status [27]. In contrast, BOLD-MRI, by quantifying renal oxygenation status, can sensitively detect changes and progression in renal function. Numerous studies have confirmed that cortical and medullary oxygenation function in patients with CKD is significantly lower than that in healthy controls, with medullary oxygenation decline serving as an effective predictor of renal function progression [28, 29]. Feng et al demonstrated that BOLD-MRI can assess early renal hypoxia in DKD, suggesting that MR2* values may be a more sensitive predictor of early DKD [18].

We found that MR2* was significantly higher than CR2* (*p* < 0.001), consistent with its physiological hypoxic characteristics and aligned with the renal oxygen supply–demand balance mechanism [20, 27]. Specifically, the high metabolic demand of the renal medulla, primarily due to active tubular reabsorption, and its relatively low blood supply result in a substantial oxygen partial pressure gradient, whereas the cortex maintains better oxygenation due to high blood flow perfusion. Furthermore, the higher MR2* in the NDRD group may be related to the primary pathological features of NDRD (mainly membranous nephropathy, 47.2%, IgA nephropathy, 21.8%, focal segmental glomerulosclerosis, and acute or chronic interstitial damage, 25.4%), among which NDRD cases involving interstitial VL accounted for 71%. Interstitial VL, inflammatory infiltration, and fibrosis exacerbate medullary hypoxia. In contrast, the lower MR2* in the DN group may be attributed to early glomerular hyperfiltration and hyperperfusion, which induce a hypoxic state in the renal medulla during the early stages of CKD (manifested as elevated medullary R2* values). However, as DN progresses, the sustained high metabolic load ultimately leads to an imbalance between oxygen demand and supply and a reversal of the cortical–medullary oxygenation gradient, resulting in improved medullary oxygenation and reduced MR2* [30–32]. In addition, the lower MR2* observed in the ESRD group may reflect reduced tubular workload and oxygen consumption. In early CKD, glomerular hyperfiltration and hyperperfusion induce medullary hypoxia, elevating R2* [28]. As DN progresses, impaired tubular concentration and sodium reabsorption, due to decreased GFR and altered tubuloglomerular feedback, reduce metabolic demand, thereby alleviating medullary hypoxia and leading to a decline in R2* [18, 33, 34] For example, Feng et al reported that diabetic patients with microalbuminuria had significantly higher MR2* than healthy controls, whereas those with macroalbuminuria showed significantly lower MR2* than the microalbuminuria group.

Previous studies on CKD have demonstrated that R2* values are closely associated with renal pathological sclerosis and tubulointerstitial fibrosis, highlighting their importance in evaluating renal histological changes [19, 20]. In this cohort, significant clinical heterogeneity was observed among the different pathological groups (DN, NDRD, and combined groups), with the DN group exhibiting the highest incidence of endpoint events (*p* < 0.001). This finding aligns with the extensive and complex pathological changes in glomerulosclerosis and interstitial fibrosis characteristic of DN [35], further corroborating the global epidemiological trend of DN as a leading cause of ESRD. Notably, the subgroup progressing to ESRD demonstrated a longer duration of diabetes, lower baseline eGFR, more severe proteinuria [23], and lower MR2* values (all *p* < 0.05), underscoring the critical importance of early identification and intervention in patients who are high-risk. CR2* and MR2* values showed weak correlations with conventional clinical indicators, such as eGFR and proteinuria. Specifically, MR2* exhibited a weak negative correlation with glomerular lesion grading (*r* = −0.28, *p* = 0.01). Medullary hypoxia may exacerbate inflammation and fibrosis through the activation of hypoxia-inducible factor pathways [36]. Although our study did not find a significant correlation between IFTA and R2*, both parameters independently predicted prognosis. The integration of BOLD-MRI with pathological indicators may optimize prognostic models, offering a novel, non-invasive tool for the precise diagnosis and treatment of DKD.

In constructing the ESRD prognostic assessment model, Cox regression analysis of the entire cohort revealed that pathological grouping, Hb, Cys-C, and MR2* were independent predictors of ESRD prognosis (all *p* < 0.05), consistent with previous reports [37]. In the DN and combined groups, MR2*, IL, and IFTA were significant in the univariate Cox regression analysis (*p* < 0.05); however, their significance decreased in the multivariate analysis (*p* > 0.05). This reduction in significance in the multivariate analysis may reflect interactions or confounding effects among variables, which can alter the individual significance of the predictors. Notably, despite the decreased significance of these indicators, the model demonstrated excellent predictive performance, with a *c*-index of 0.91, suggesting that these indicators may still hold clinical relevance after controlling for confounding factors [38–40]. The integration of multimodal data enables more precise risk stratification, facilitating the identification of high-risk patients and optimizing therapeutic decision-making.

We acknowledge that the independent contribution of MR2* is indeed modest compared to DR. As shown in Supplementary Fig. 1, MR2* demonstrated a significant association in univariate analysis (OR 0.87, *p* = 0.022), but the effect attenuated in multivariate analysis (OR 0.94, *p* = 0.571). This attenuation primarily reflects the strong predictive power of DR (OR 31.13, *p* < 0.001), which captures overlapping microvascular information. Importantly, the effect direction remained consistently protective (OR < 1) with minimal magnitude change, supporting the biological validity of MR2* as a quantitative marker of renal medullary oxygenation status. Similarly, in Fig. 6, the AUC values for MR2* alone were moderate (0.610–0.641). However, this moderate degree of independent contribution is in line with clinical expectations and does not reduce the research value of BOLD-MRI. Because MR2* is a non-invasive and potentially valuable imaging biomarker, it contributes meaningful research value to the comprehensive assessment of DN. For the diagnostic model, we used feature importance to identify discriminative parameters. The machine learning–based diagnostic model (RF: AUC = 0.901) outperformed traditional logistic regression (AUC = 0.876), highlighting its superiority in handling complex clinical data and feature selection [41].

This exploratory study on the diagnostic and prognostic value of BOLD-MRI in DKD patients with renal biopsy provides a basis for clinical decision-making. However, considering the cost and clinical benefits of BOLD-MRI, MR2* may be used as a supplementary tool in specific cases (such as when other biomarkers are not applicable or are insufficiently accurate). This study also has several limitations. First, the single-center design may restrict the generalizability and extrapolation of the findings, necessitating further validation through multicenter studies with larger sample sizes. Second, BOLD-MRI signals can be influenced by various physiological factors, including respiratory artifacts, blood pH, temperature, red blood cell volume, hydration status, and salt intake [42]. To mitigate these potential confounders, several measures were implemented in this study: a fasting period of at least 4 h was required before imaging, images with compromised quality were excluded, and a double-blind approach was adopted for data processing. Additionally, machine learning methods were employed during data analysis and modeling to control for confounding factors.

The median follow-up in this study was 15 months, indicating that longer observation will be required to fully define the long-term prognosis. Future research should focus on multicenter longitudinal cohort studies to validate the generalizability of the model, explore the combined application of BOLD-MRI with emerging biomarkers, and develop AI-driven radiomics models to enhance diagnostic sensitivity and specificity. These efforts will contribute to refining the precision medicine framework for DKD. BOLD-MRI provides a novel non-invasive tool for risk stratification and individualized intervention in DKD.

## Conclusion

BOLD-MRI technology, particularly MR2*, shows potential value as a non-invasive diagnostic and prognostic tool in the assessment of DKD. However, BOLD-MRI remains a promising, yet exploratory, technique that requires external validation and interventional studies before clinical implementation.

## Supplementary information

ELECTRONIC SUPPLEMENTARY MATERIAL

## Data Availability

The datasets generated and analyzed during the present study are available from the corresponding author upon reasonable request.

## Abbreviations

24hUPro: 24-h urine protein
AH: Arteriolar hyalinosis
Alb: Albumin
AS: Arteriosclerosis
AUC: Area under the curve
BMI: Body mass index
BOLD-MRI: Blood–oxygen-level-dependent magnetic resonance imaging
BUN: Blood urea nitrogen
CKD: Chronic kidney disease
CR2*: Cortical R2*
Cys-C: Cystatin C
DKD: Diabetic kidney disease
DN: Diabetic nephropathy
DR: Diabetic retinopathy
eGFR: Estimated glomerular filtration rate
ESRD: End-stage renal disease
GC: Glomerular classification
Hb: Hemoglobin
HbA1c: Glycosylated hemoglobin
IFTA: Interstitial fibrosis and tubular atrophy
II: Interstitial inflammation
IL: Interstitial lesions
LDL: Low-density lipoprotein
MR2*: Renal medullary R2*
NDRD: Non-diabetic renal disease
RF: Random forest
SBP: Systolic blood pressure
Scr: Serum creatinine
T2DM: Type 2 diabetes mellitus
TC: Total cholesterol
TG: Triglycerides
VL: Vascular lesions
