# Supplementary material for: Clinical utility of BOLD-MRI in accurate diagnosis and prognostic evaluation of diabetic nephropathy: a prospective renal biopsy-based cohort study
Source: Insights Imaging. 2026 Apr 20;17:106. doi: 10.1186/s13244-026-02274-9 (PMC13096298; doi:10.1186/s13244-026-02274-9)
Supplement: Supplementary file 1 — ELECTRONIC SUPPLEMENTARY MATERIAL [file 13244_2026_2274_MOESM1_ESM.pdf]

Clinical Utility of BOLD-MRI in Accurate Diagnosis and Prognostic Evaluation of Diabetic Nephropathy: A Prospective Renal Biopsy-Based Cohort Study.

ELECTRONIC SUPPLEMENTARY MATERIAL

Supplementary Table 1. BOLD-MRI correlation coefficients (ICCs) results

Intraclass Correlation Coefficient of cortex R2*

	Intraclass Correlation[b]	95% Confidence Interval		F Test with True Value 0			
		Lower Bound	Upper Bound	Value	df1	df2	Sig
Single Measures	.965[a]	0.943	0.978	55.713	65	65	0
Average Measures	0.982	0.971	0.989	55.713	65	65	0

Two-way random effects model where both people's effects and measurement effects are random.
[a] The estimator is the same, whether the interaction effect is present or not.
[b] Type A intraclass correlation coefficients using an absolute agreement definition.

Intraclass Correlation Coefficient of medulla R2*

	Intraclass Correlation[b]	95% Confidence Interval		F Test with True Value 0			
		Lower Bound	Upper Bound	Value	df1	df2	Sig
Single Measures	.978[a]	0.964	0.986	88.338	65	65	0
Average Measures	0.989	0.982	0.993	88.338	65	65	0

Two-way random effects model where both people's effects and measurement effects are random.
[a] The estimator is the same, whether the interaction effect is present or not.
[b] Type A intraclass correlation coefficients using an absolute agreement definition.

Supplementary Table 2. Distribution of missing data

Variable	Missing.count
Male	0
Hypertension	1
BMI	0
Diabetes duration	0
DR	0
SBP	0
DBP	0
24h UPro	0
Gh	0
FBG	0
BUN	0
Alb	0
P	7
HbA1c	9
Scr	0
eGFR	0
TC	1
Na	7
LDL	3
Hb	0
HCT	8
cortex R2*	0
medulla R2*	0

Supplementary Table 3. Variance inflation factor (VIF) of prognostic model variables

Prognostic model variables	VIF
24h UPro	3.224568922
eGFR	2.185096255
Hb	1.581727493
Medulla R2*	1.200894606
Glomerular classification of DN	2.255265193
Interstitial lesions	4.957923388
IFTA	5.675899112

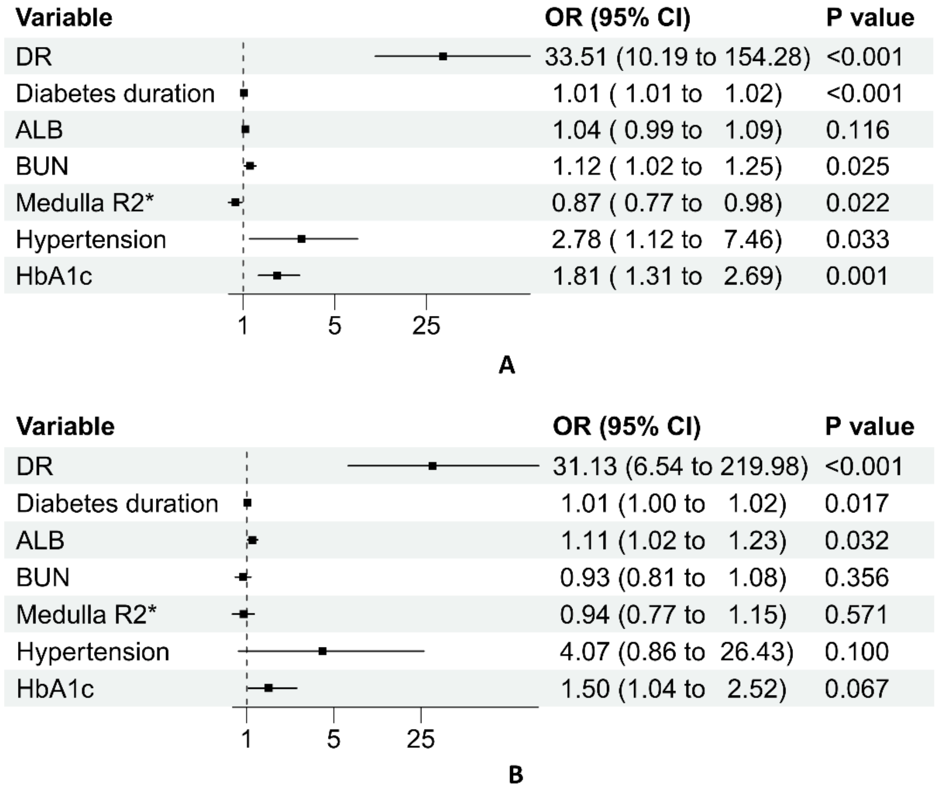

Supplementary Figure 1. Univariate and Multivariate Logistic Regression Analysis for Differential Diagnosis

(A).Univariate analysis.

(B).Multivariate analysis.
